# Modeling Blast Crisis Using Mutagenized Chronic Myeloid Leukemia-Derived Induced Pluripotent Stem Cells (iPSCs)

**DOI:** 10.3390/cells12040598

**Published:** 2023-02-12

**Authors:** Jusuf Imeri, Christophe Desterke, Paul Marcoux, Gladys Telliam, Safa Sanekli, Sylvain Barreau, Yucel Erbilgin, Theodoros Latsis, Patricia Hugues, Nathalie Sorel, Emilie Cayssials, Jean-Claude Chomel, Annelise Bennaceur-Griscelli, Ali G. Turhan

**Affiliations:** 1 INSERM UMR-S-1310, Université Paris Saclay, 94800 Villejuif, France and ESTeam Paris Sud, Université Paris Saclay, 94800 Villejuif, France; 2 APHP Paris Saclay, Department of Hematology, Hôpital Bicêtre & Paul Brousse, 94800 Villejuif, France; 3 Aziz Sancar Institute of Experimental Medicine, Istanbul University, 34093 Istanbul, Turkey; 4 Service de Cancérologie Biologique, CHU de Poitiers, 86000 Poitiers, France; 5 Service d’Oncologie Hématologique et Thérapie Cellulaire, CHU de Poitiers, 86021 Poitiers, France; 6 INGESTEM National iPSC Infrastructure, 94800 Villejuif, France; 7 CITHERA, Centre for iPSC Therapies, INSERM UMS-45, Genopole Campus, 91100 Evry, France

**Keywords:** iPSC, blast crisis CML, CML modeling, single-cell transcriptomics, CD25

## Abstract

Purpose: To model CML progression in vitro and generate a blast crisis (BC-CML) model in vitro in order to identify new targets. Methods: Three different CML-derived iPSC lines were mutagenized with the alkylating agent ENU on a daily basis for 60 days. Cells were analyzed at D12 of hematopoietic differentiation for their phenotype, clonogenicity, and transcriptomic profile. Single-cell RNA-Seq analysis has been performed at three different time points during hematopoietic differentiation in ENU-treated and untreated cells. Results: One of the CML-iPSCs, compared to its non-mutagenized counterpart, generated myeloid blasts after hematopoietic differentiation, exhibiting monoblastic patterns and expression of cMPO, CD45, CD34, CD33, and CD13. Single-cell transcriptomics revealed a delay of differentiation in the mutated condition as compared to the control with increased levels of *MSX1* (mesodermal marker) and a decrease in *CD45* and *CD41*. Bulk transcriptomics analyzed along with the GSE4170 GEO dataset reveal a significant overlap between ENU-treated cells and primary BC cells. Among overexpressed genes, *CD25* was identified, and its relevance was confirmed in a cohort of CML patients. Conclusions: iPSCs are a valuable tool to model CML progression and to identify new targets. Here, we show the relevance of CD25 identified in the iPSC model as a marker of CML progression.

## 1. Introduction

Chronic myeloid leukemia (CML) is characterized by the presence of the *BCR::ABL1* fusion gene generated by the reciprocal translocation between chromosomes 9 and 22, t(9;22) (q34.1;q11.2). The BCR::ABL fusion protein generated from this translocation is highly oncogenic and characterized by an enhanced tyrosine kinase (TK) activity [1]. 

CML is a relatively rare disease with an incidence of 1/ and a median age at diagnosis of 55 years. The incidence is higher in males as compared to females (ratio: 1.3–1.5/1) [2].

CML is the prototype of a clonal malignancy of the hematopoietic cell [3]. The disease is initiated by the appearance of the hallmark of the disease, the Philadelphia (Ph1) chromosome in a hematopoietic stem cell, which is followed by the acquisition of novel abnormalities leading to clonal progression, accelerated phase (AP), and blast crisis (BC) [4]. 

The frequency of this progression has now been considerably reduced since the introduction of tyrosine kinase inhibitors (TKIs) to therapy, but BC-CML still occurs, albeit at low frequency and remains of dismal prognosis even in the era of TKIs [5], potentially due resistance to TKIs and to clonal selection [5,6]. Using serially collected patient samples, seminal and now classical work has established the main cytogenetic and molecular events observed during the progression of the disease towards AP-CML and eventually BC-CML. These include cytogenetic abnormalities identified as “major” and “minor” routes [7,8].

Several works performed using patient samples and models have also identified, during the last two decades, major molecular events involved in this progression, such as TP53 mutations [9]. BCR-ABL-expressing cells are also prone to develop a mutator phenotype [10], which could be due to the presence of increased oxidative stress [11], which leads to clonal selection under therapy [12]. CML cells are finally doomed by the deficiency of several DNA repair mechanisms involving DNA-PKcs [13] and BRCA1 [14], as well as abnormalities of specific DNA repair genes such as BLM [15] and other repair mechanisms such as NER [16] or MMR [17]. 

It is now established that CML leukemic stem cells (CML-LSCs) are resistant to TKI [18,19,20]. These cells are difficult to isolate, and despite recent work suggesting the expression of some markers predominantly on them [21], there are no specific biomarkers universally accepted for CML-LSCs [22,23]. Moreover, current in vitro and in vivo models do not recapitulate the evolution of human CML, especially with regard to the first stages of the disease and its natural evolution. We and others have previously shown the potential of the use of CML patient-derived induced pluripotent stem cells (iPSCs) for CML modeling [24,25]. These models can recapitulate some aspects of CML, such as the myeloproliferative phenotype, but they do not allow the generation of a dynamic in vitro model of CML progression. This progression occurs through several genomic events over several years in CML patients, and the serial analysis of these events in individual cases is not possible. Patient-specific CML-iPSCs exhibit the potential of recapitulating this clonal progression as they can be expanded indefinitely at a pluripotent stage and induced for hematopoietic differentiation using in vitro techniques. We have previously shown that the genomic instability inherent to CML leukemic cells can be induced by in vitro mutagenesis by the use of N-ethyl-N-nitrosourea (ENU). This allows the generation of genomic instability and could therefore enable the dynamic modeling of CML evolution [26]. 

Here, we have designed a strategy to mimic AP/BP in CML patients by inducing in vitro mutagenesis. Applied to three CML-derived iPSCs, we show that this technology can be used to mimic cytological and genomic instability patterns observed in primary blast crisis cells, including the overexpression of *IL2RA/CD25*, that have been validated in a cohort of primary CML patients in AP/BC-CML.

## 2. Materials and Methods

### 2.1. Patients 

Table 1 shows the clinical characteristics of the three CML patients and their corresponding iPSCs included in this study. CD34+ cells from diagnostic (UPN-27 and UPN-32) and from AP-CML samples (UPN-34) have been used to generate iPSCs [27,28]. This non-clinical study has been approved by the INSERM Ethical committee. PB33 control cells were generated from hematopoietic cells of a healthy donor. All patients and the healthy donor (42/M) gave their informed consent in accordance with the Declaration of Helsinki.

### 2.2. iPSC Cultures

iPSCs were cultured either in feeder-free or in classical stroma-supported murine embryonic fibroblast (MEF) feeder conditions. Feeder-free cultures were performed in Geltrex coated dishes (ThermoFisher Scientific, Illkirch, France, A1413201) and fed daily with Essential 8 flex Medium (ThermoFisher Scientific, A2858501). iPSCs were passaged twice a week in aggregates with EDTA dissociation. Alternatively, they were maintained on mitomycin-C-inactivated Mouse Embryonic Fibroblasts (MEF) feeder cells with DMEM KnockOut (Gibco, Illkirch, France, 10829018) supplemented with 10% Knock-Out Serum Replacement (Gibco, Illkirch, France, 10828010), Glutamax (1X) (Gibco, Illkirch, France, 35050061), Penicillin/Streptomycin (100 U/mL) (Gibco, Illkirch, France, 15140122), β-mercaptoethanol, and basic FGF (0.1 mg/mL) (Miltenyi Biotec, Paris, France, 130-093-840). For passaging iPSCs cultured on MEFs, cells were incubated with collagenase (10 mg/mL) (Fisher Scientific, Illkirch, France, 10780004) for 5–7 min at 37 °C, 5% CO_2_, and the appropriate amount of aggregates was used to seed new MEF-coated dishes.

### 2.3. Teratoma Assays 

Teratoma assays were performed by injecting 1 × 10^6^ iPSCs in NOD SCID gamma (NSG) mice. Cells were mixed with Matrigel (150 μL of Matrigel for 1 × 10^6^ iPSCs) and injected in the right hind leg of the mouse. A total of 10 weeks after injection, mice were sacrificed, and pathological analysis of teratomas was performed.

### 2.4. N-ethyl-N-Nitrosourea-(ENU) Induced Mutagenesis 

CML-iPSCs were cultured in MEFs in the presence or absence of ENU (10 µg/mL) for 60 days with daily addition of the drug in cultures. They were then characterized using pluripotency and genomic instability markers and were adapted to feeder-free conditions needed for further experiments.

### 2.5. Karyotyping 

Karyotype analyses were performed using cell pellets collected at different time points using standard methods as described previously [25]. 

### 2.6. Hematopoietic Differentiation from iPSCs before and after Mutagenesis

Hematopoietic differentiation of iPSCs has been performed using a STEMDiff hematopoietic kit (STEMCELL Technologies, Grenoble, France, 05310) according to the manufacturer’s recommendations. Briefly, iPSCs (cultured on Geltrex with E8-flex medium) have been dissociated in aggregates of 50–100 μm by using EDTA (0.5 mM). A total of 50 aggregates have been seeded per well in a 12-well cell culture plate (Corning, Hazebrouck, France, 3513) coated with Geltrex. The medium was changed according to the manufacturer’s instruction, and cells were harvested on day 12 of hematopoietic differentiation using TrypLE select (ThermoFisher Scientific, Illkirch, France, 12604021). For single-cell transcriptomic assays, cells were harvested on day +5, day +9, and day +13 of hematopoietic differentiation.

### 2.7. Evaluation of Cytological Characteristics of Cells before and after Mutagenesis

Cells collected from the hematopoietic cultures on days 12–14 of differentiation were analyzed after May–Grünwald–Giemsa staining. Approximately 10^5^ cells were washed twice with PBS. Cytospins were performed on slides using a Cytospin 4 centrifuge (ThermoFisher Scientific, Illkirch, France). Thereafter, slides were air-dried for 25 min and stained with RAL Kit 555 (RAL Diagnostics, Martillac, France, 361550-0000). The slides were analyzed on a Nikon Eclipse 90i microscope (Champigny sur Marne, France), and images were taken with a Nikon camera DS-Fi1 and NIS-Elements V. 5.20 software. 

### 2.8. Flow Cytometry

Cells collected from day +12 of hematopoietic differentiation were counted in trypan blue to determine their viability and stained with the following antibodies (Table 2) in PBS at 4 °C for 20 min. 

Cells were thereafter washed and resuspended in PBS with 1 μg/mL 7-Aminoactinomycin D (7-AAD) (Sigma-Aldrich, Saint-Quentin-Fallavier, France, 7240-37-1). Stained cells were analyzed with a BD LSRFortessa^TM^ (BD Biosciences, San Jose, CA, USA,) flow cytometer and FlowJo analysis software. 

### 2.9. Clonogenic Assays

Non-adherent cells collected at day +12 of hematopoietic differentiation were counted and plated in methylcellulose-based medium (MethoCult^TM^ H4434, STEMCELL Technologies, Grenoble, France) at the concentration of cells/mL and incubated for 14 days in a 37 °C incubator with 5% CO_2_. On day +14, colonies were enumerated.

### 2.10. Long-Term Culture Initiating Cell (LTC-IC) Assays 

LTC-IC assays have been performed according to previously reported techniques [19]. Briefly, cells collected at day +12 of hematopoietic differentiation were counted and started on long-term culture assays in triplicates using 4.5 × 10^4^ cells/dish. Cultures were maintained at 33 °C on MS-5 stromal cells with weekly half-medium changes (MyeloCult^TM^ H5100, STEMCELL Technologies). At week +5, non-adherent and adherent cells were collected, counted, and plated in methylcellulose (MethoCult^TM^ H4434, STEMCELL Technologies, Grenoble, France) at the concentration of 5 × 10^3^ cells/dish in triplicates. The number of clonogenic growth was evaluated at days +14 and +21.

### 2.11. Evaluation of Ruxolitinib and Imatinib Sensitivity of PB34 and PB34-ENU Hematopoietic Cells 

Hematopoietic cells collected from day +12 of differentiation cultures were tested on clonogenic assays with and without TKIs. For each condition, 1 × 10^4^ cells were plated in methylcellulose (MethoCult^TM^ H4434, STEMCELL Technologies) with a TKI drug (alone or in combination) at the following concentrations: 1 μM Imatinib (Sigma-Aldrich, SML1027) and 1 μM Ruxolitinib (Selleckchem, Planneg, Germany, S1378). The number of clonogenic growth was evaluated at day +14. All experiments were performed in triplicates.

### 2.12. Patients and Healthy Donors

*CD25* mRNA expression was assessed by qRT-PCR on blood samples from 25 patients diagnosed with CP-CML and 14 patients with BC-CML. These experiments were simultaneously performed on the bone marrow of patients with acute myeloid leukemia (AML) and B-cell lymphoblastic leukemia (B-ALL) at diagnosis (Table 3). For acute leukemias, bone marrow aspirates were obtained from each patient at diagnosis. No additional samples were collected for this study. A cohort of 15 healthy donors was also used as a control. Seven patients were analyzed at diagnosis (CP-CML) and after the leukemic transformation (BC-CML). All patients and healthy donors provided informed consent in accordance with the declaration of Helsinki.

### 2.13. Quantitative RT-PCR Assays

Total RNA from whole blood or bone marrow samples was reverse transcribed using the High-Capacity cDNA Reverse Transcription Kit (Life Technologies, Foster City, CA, USA), and qRT-PCR experiments were performed using the StepOnePlus real-time PCR system (Life Technologies, Foster City, CA, USA). TaqMan pre-developed assays reagent (Life Technologies, Foster City, CA, USA) were used to quantify *CD25* (Hs00907777_m1) mRNA transcripts. *CD25* expression was normalized by measuring *ABL1* mRNA levels in the same cDNA samples (internal reference). PCR reactions were prepared in duplicates in a final volume of 25 μL using the TaqMan Universal PCR Master Mix (Life Technologies, Foster City, CA, USA). The *CD25/ABL1* percentage was established using the DCt method.

### 2.14. Single-Cell RNA-Sequencing of Mutagenized and Unmutagenized CML-iPSC-Derived Hematopoietic Cells 

Non-adherent cells obtained from the hematopoietic induction cultures were used for this experiment performed on PB32 cells and their counterparts after ENU mutagenesis (PB32-ENU). Cells from day +5, day +9, and day +13 of hematopoietic differentiation were harvested. Three triplicate wells were independently collected for each time point, and cells were counted and further processed using the Chromium10× Genomics® protocol (V3.1). Single-cell Gel Bead-In-EMulsions (GEMs) were generated using a Chromium Controller instrument (10× Genomics). Sequencing libraries were prepared using Chromium Single Cell 3′ Reagent Kits (10× Genomics), according to the manufacturer’s instructions. Briefly, GEM-RT was performed in a thermal cycler using a protocol including incubation at 53 °C for 45 min and 85 °C for 5 min. Post-GEM-RT Cleanup using DynaBeads MyOne Silane Beads was followed by cDNA amplification (98 °C for 3 min, cycled 12 × 98 °C for 15 s, 67 °C for 20 s, 72 °C for 1 min, and 72 °C for 1 min). After a cleanup with SPRIselect Reagent Kit and fragment size estimation with High Sensitivity™ HS DNA kit run on 2100 Bioanalyzer (Agilent Technologies, Santa Clara, CA, USA), the libraries were constructed by performing the following steps: fragmentation, end-repair, A-tailing, SPRIselect cleanup, adaptor ligation, SPRIselect cleanup, sample index PCR, and SPRIselect size selection.

The fragment size estimation of the resulting libraries was assessed with High Sensitivity™ HS DNA kit run on 2100 Bioanalyzer (Agilent Technologies, Santa Clara, CA, USA) and quantified using the Qubit™ dsDNA High Sensitivity HS assay (ThermoFisher Scientific, USA). Libraries were then sequenced by pair with a HighOutput flowcell using an Illumina Nextseq 500 with the following mode: 26 bp (10× Index + UMI), 8 bp (i7 Index), and 57 bp (Read 2).

The sequencing data were processed into transcript count tables with the Cell Ranger Single Cell Software Suite 1.3.1 by 10× Genomics (http://10xgenomics.com/, accessed on 9 February 2023). Raw base call files from the Nextseq 500 were demultiplexed with the cellranger mkfastq pipeline into library-specific FASTQ files. The FASTQ files for each library were then processed independently with the cellranger count pipeline. This pipeline used STAR to align cDNA reads to the Mus musculus transcriptome (sequence: GRCm38, annotation: Gencode v25). Once aligned, barcodes associated with these reads—cell identifiers and Unique Molecular Identifiers (UMIs)—underwent filtering and correction. Reads associated with retained barcodes were quantified and used to build a transcript count table. The resulting data for each sample were then aggregated using the cellranger aggr pipeline, which performed a between-sample normalization step and concatenated the two transcript count tables. 

For each of the 6 experimental conditions after sequencing, fastq files were mapped on GRChg38 human genome processed for demultiplexing with cellranger software version 4.0.0. h5 filtrated outfiles for each experiment were processed individually in R software environment version 4.2.1 (10× Genomics, San Francisco, CA, USA) with Seurat R-package version 4.1.1 (Paul Hoffman, Satija Lab and Collaborators, New York, NY, USA) [29]. Single-cell processing was performed individually by filtrating cells expressing less than one hundred transcripts and also removing transcripts expressed in less than 3 cells. Before integration, the six experiments were individually log normalized, and integration was based on searching common anchors on the twenty-first mathematical dimensions between experiments. After integration, the combined object composed of 63,462 cells and of 28,182 features was scaled. Successive dimension reductions by principal component analysis and t-distributed stochastic neighbor embedding were performed to identify cell communities within a graph structure; for example, a K-nearest neighbor (KNN) graph approach [30]. Experiments/cell clusters proportion barplot was drawn with ggplot2 R-package version 3.3.6 [31].

### 2.15. Exome Analysis of Mutagenized CML-iPSCs

To determine genetic changes induced by ENU-induced mutagenesis and their stability, we have performed, in the PB32 and PB32-ENU cell line, an exome sequencing analysis. Library preparation, exome capture, sequencing, and data analysis have been performed by IntegraGen SA (Evry, France). Genomic DNA was captured using Agilent in-solution enrichment methodology (SureSelect XT Clinical Research Exome, Agilent Technologies, Santa Clara, CA, USA) with their biotinylated oligonucleotides probes library (SureSelect XT Clinical Research Exome—54 Mb, Agilent Technologies), followed by paired-end 75 bases massively parallel sequencing on Illumina HiSeq4000. Sequence capture, enrichment, and elution were performed according to the manufacturer’s instructions and protocols (SureSelect, Agilent technologies, Les Ulis, France) without modification except for library preparation performed with NEBNext^®^ Ultra kit (New England Biolabs^®^, Evry-Courcouronnes, France). For library preparation, 600 ng of each genomic DNA was fragmented by sonication and purified to yield fragments of 150–200 bp. Paired-end adaptor oligonucleotides from the NEB kit were ligated on repaired, tailed fragments, then purified and enriched by 8 PCR cycles. An amount of 1200 ng of these purified libraries were then hybridized to the SureSelect oligo probe capture library for 72 h. After hybridization, washing, and elution, the eluted fraction was PCR-amplified with 9 cycles, purified, and quantified by qPCR to obtain a sufficient DNA template for downstream applications. Each eluted, enriched DNA sample was then sequenced on an Illumina HiSeq4000 as paired-end 75b reads. Image analysis and base calling were performed using Illumina Real Time Analysis (2.7.7, Illumina, San Diego, CA, USA) with default parameters.

### 2.16. Bulk RNA Transcriptomics of CML-iPSC-Derived Hematopoietic Cells before and after Mutagenesis

RNA was extracted from the hematopoietic cells derived from the three mutagenized and unmutagenized CML-iPSCs. Samples were processed in duplicate for performing hybridization of Clariom S assay human (ThermoFisher Scientific, USA). Robust microarray analysis (RMA) normalization [32] was performed in Transcriptome Analysis Console (TAC) software (ThermoFisher Scientific, USA), followed by differential expressed gene analysis with limma algorithm [33] between ENU and control conditions of the same individual iPSC. Gene signatures of the three distinct iPSCs were compared by barplot and Venn diagram. Upregulated genes by ENU in PB32 iPSCs were used as a predictive signature of the circulating hematopoietic progenitor of CML patients in blast crisis as compared with ones from patients in chronic phase (Geodataset GSE4170) [34]. This machine learning analysis was performed with pamr R-package version 1.56.1 by leaving one out cross-validation process [35]. ENU-upregulated signature was also used as a geneset during geneset enrichment analysis (GSEA) [36]. Unsupervised principal component analysis was performed with FactoMineR R-package version 2.5 [37]. Functional enrichment was performed with the Toppgene web [38] application on Gene Ontology cellular component database [39]. A functional enrichment network was drawn with Cytoscape standalone application version 3.6.0 (Cytoscape Consortium, San Diego, CA, USA) [40]. Expression heatmap was drawn with pheatmap R-package version 1.0.12 (Raivo Kolde, Boston, MA, USA).

## 3. Results

### 3.1. Generation and Characterization of Mutagenized CML-iPS Cells

CML-iPSCs from three patients were generated as previously described [27,28] (Table 1). Briefly, reprogramming was realized by using a Sendai kit for either purified PB CD34+ cells for PB27 and PB32 or leukemic PBMC for PB34. To induce mutagenesis, reprogrammed cells from similar passages were treated with ENU for 60 days (Supplementary Figure S1A). During this period of ENU treatment, there were no significant morphological differences detectable in the iPSC colonies. At day +60, ENU was removed from the cultures. Mutagenized iPSCs have been characterized for the induction of genetic instability markers as well as for their pluripotency. The genomic instability has been evaluated by staining by Western blotting the phospho-γH2AX in iPSCs after 30 and 60 days of ENU treatment. An increase in the phospho-γH2AX was observed for all CML-iPSCs after ENU treatment, suggesting that ENU induces genomic instability (Supplementary Figure S1B). 

**Figure 1:**
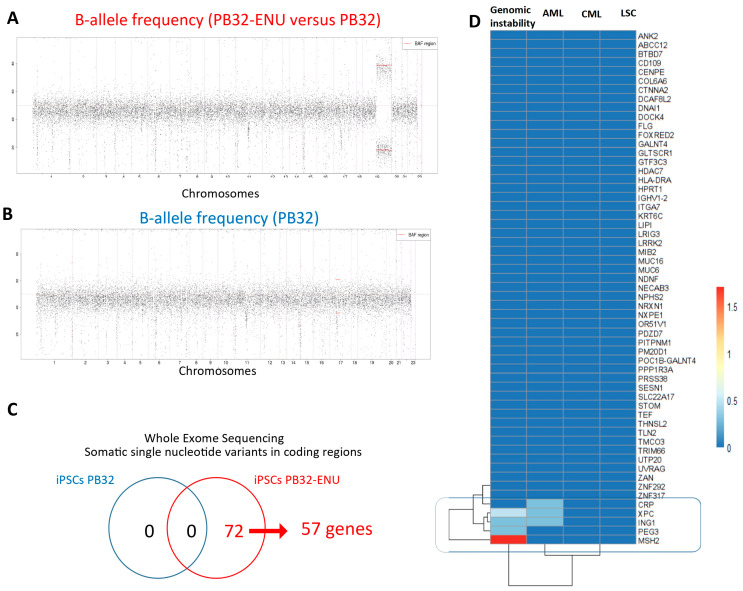
Whole exome sequencing of PB32 in the context of ENU treatment. (**A**) B-allele frequency plot all over human autosomes of PB32. (**B**) B-allele frequency plot all over human autosomes comparing PB32-ENU versus PB32. (**C**) Venn diagram of single nucleotide variations (SNV) found in PB32-ENU versus PB32. (**D**) Pubmed text mining citation heatmap highlighting genes affected by SNV and known to be implicated in genomic instability-related functions.

To determine the mutagenic events generated by ENU treatment of CML-iPSCs, we have performed exome sequencing in the PB32 cell line as compared to its ENU-mutagenized counterparts. The goal of this experiment was also to determine the stability of the mutagenic changes observed at the genomic level. As shown in Figure 1A, a large unidisomy affecting the long arm of chromosome 19 was observed in the PB32-ENU condition (Figure 1A) as compared to the PB32 condition (Figure 1B). The calling of somatic variants was carried out for each of the conditions. After filtration of rare somatic variants (no synonymous missense and stop gained), no variants were found in the condition PB32 (Figure 1C), but 72 variants were identified in the PB32-ENU (Figure 1C). These 72 somatic single nucleotide variants were found to be localized in 57 gene loci across the whole exome (Figure 1C). Pubmed text mining performed on these mutated genes highlighted the importance of *MSH2*, *PEG3*, and *ING1* in genomic instability and that of *ING1*, *XPC*, and *CRP* in AML. In particular, it is of interest to note that XPC (Xeroderma Pigmentosum group C) is a DNA repair protein interacting with the NER (nucleotide excision repair) mechanism and could play a role in the pathogenesis of CML via its genetic polymorphisms [41,42]. Interestingly, another xeroderma pigmentosum protein (XPB) has previously been reported to interact directly with BCR::ABL [43]. However, for the remaining genes, there are no publications relating them to genomic instability, AML, CML, or LSC (Figure 1D).

The pluripotency of the mutagenized clones was then evaluated by using FACS analysis and teratoma assays. As can be seen in Supplementary Figure S2A–D, mutagenesis did not alter the expression of SSEA4, Tra1–60, and OCT4 in all cell lines. In teratoma assays, all cell lines were able to generate in vivo the three embryonic layers (Supplementary Figure S2A–D). No additional cytogenetic abnormalities were detectable in PB34 and PB27, although some cytogenetic changes were documented previously in the PB32-ENU cell line [26] at a karyotypic level after ENU treatment. All analyzed clones were Ph1+ (Supplementary Figure S2A–D).

### 3.2. Induction of Hematopoietic Differentiation from ENU-Mutagenized and Unmutagenized iPSCs

**Figure 2:**
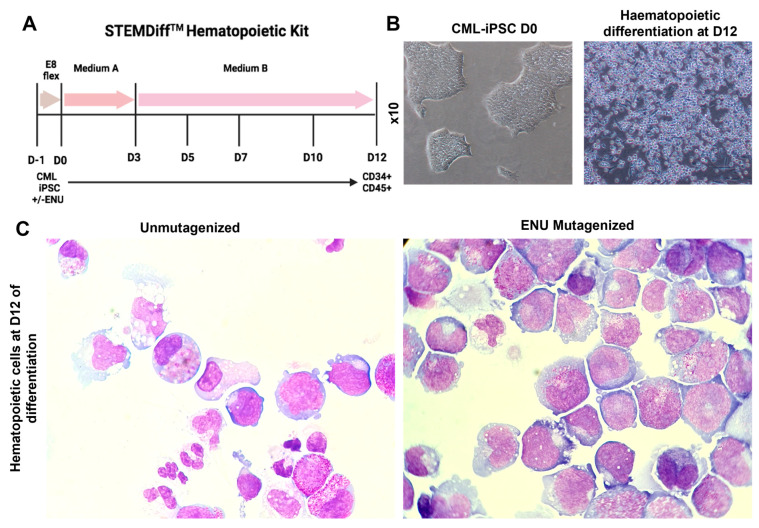
Hematopoietic differentiation of mutagenized and unmutagenized CML-iPSC. (**A**) Hematopoietic differentiation protocol of mutagenized and unmutagenized CML-iPSCs. During the first three days, the generation of mesoderm was followed by hematopoietic induction from D3 to D12. (**B**) Morphology of CML-iPSCs at their pluripotent stage (**left**) and at D12 of differentiation (**right**). (**C**) Microscope pictures (magnification ×60) of May–Grünwald and Giemsa staining at day 12 of floating hematopoietic cells differentiated from unmutagenized CML-iPSC (**left**) and mutagenized CML-iPSC (**right**).

We induced hematopoietic differentiation of mutagenized and non-mutagenized iPSCs by using the STEMdiff hematopoietic kit (STEMCELL Technologies) (Figure 2A). Cultures from three CML-iPSCs, as well as their mutagenized counterparts, were evaluated daily for morphological changes. These cultures initially showed a complete adhesion of iPSC-derived cells to the dishes with no evidence of cells in the non-adherent fraction. Starting day +5, we observed the appearance of budding cells with expulsion to the non-adherent fraction of round cells, which gradually increased in numbers (Figure 2B). No-adherent cells obtained on days 12–14 were then analyzed by May–Grünwald–Giemsa (MGG) staining. As can be seen in Figure 2C, there was a major difference in cytological features between cultures with and without ENU-induced mutagenesis, as in ENU-treated cultures, there was a clear arrest of differentiation. This difference was highly significant in PB32-ENU condition with a generation of blast cells with cytological features of monoblasts (Figure 2C), whereas hematopoietic cells from non-mutagenized cells exhibited a persistent differentiation with the presence of metamyelocytes and polynuclear neutrophils. Blast cells grew for several weeks in MS-5 stromal cultures in the presence of growth factors (IL-3, Flt-3L, TPO, and SCF), but did give rise to permanently growing cell lines.

The induction of blast phenotype was highly prominent in the PB32 cell line, but in the PB34 cells after mutagenesis, some arrest of differentiation has also been documented cytologically (Supplementary Figure S3). Despite the fact that there was a clear difference between PB27 cells with or without mutagenesis, mutagenized PB27 cells (See below) did not generate a cytologically detectable blast cell phenotype (data not shown).

### 3.3. Characterization of Mutagenized Hematopoietic Cells Using Flow Cytometry

**Figure 3:**
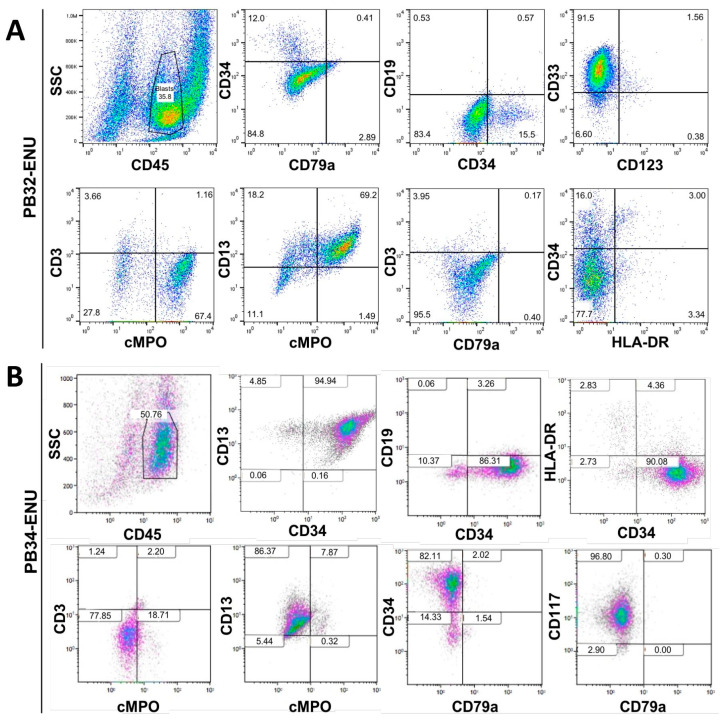
Phenotypic characterization of in vitro generated blasts. FACS staining for myeloid and lymphoid markers of PB32-ENU (**A**) and PB34-ENU (**B**) cells at day 13 of hematopoietic differentiation.

At day +13 of hematopoietic differentiation, cells were characterized by flow cytometry for different surface markers. Their phenotype analysis showed a typical myeloid profile with an expression of CD45, CD34, cMPO+, CD33+, and CD13+, with the absence of CD19, CD3, and CD79a confirming the myeloid engagement of cells (Figure 3A,B). Only about 15% of PB32-ENU cells were CD34+, while 90% of PB34-ENU were positive for this marker. Despite the major cytological differences obtained in the mutagenized PB32 cells, phenotypically, there was one little difference between mutagenized and unmutagenized hematopoietic cells, with slightly reduced expression of cMPO in the latter (Supplementary Figure S4). PB34-ENU-derived cells were highly positive for c-Kit (CD117). Both cell lines were negative for HLA-DR (Figure 3A,B).

### 3.4. Long-Term ENU Exposure Induces an Enhancement of CML-iPSC Hematopoiesis as Compared to Normal iPSC-Derived Clonogenic Activity 

**Figure 4:**
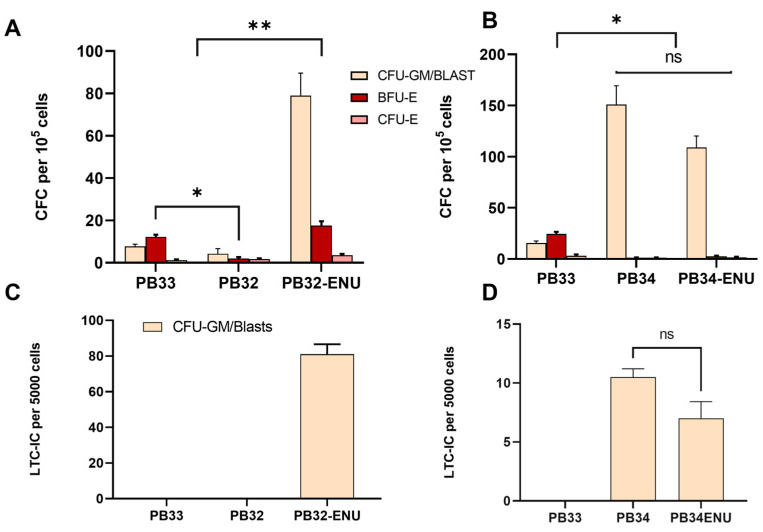
CFC and LTC-IC assays for mutated and unmutated cells at D13 of hematopoietic differentiation. (**A**) CFC assays for PB33 (WT, no BCR::ABL), PB32, and PB32-ENU according to the number of colonies per 10^5^ cells. (**B**) LTC-IC for PB33, PB32, and PB32-ENU according to the number of colonies per 5000 cells. (**C**) CFC assays for PB33, PB34, and PB34-ENU according to the number of colonies per 10^5^ cells. (**D**) LTC-IC for PB33, PB34, and PB34-ENU according to the number of colonies per 5000 cells. All experiments have been performed in triplicates. The total number of colonies was compared, and P-values were calculated using a two-tailed Student’s *t*-test. ns, not significant; *, *p* < 0.05; **, *p* < 0.01.

Colony-forming cell (CFC) assays have been performed for PB32, PB34, their mutagenized counterparts, and PB33 (control iPSC) after hematopoietic differentiation. At day +14 of hematopoietic differentiation, non-adherent cells were plated in methylcellulose. Colonies were enumerated 14 days after. A major increase in the number of colonies was observed in the PB32-ENU condition as compared to the PB32 and the control condition (Figure 4A). As can be seen in this figure, non-mutagenized PB32 generated almost no clonogenic activity, whereas in its mutagenized counterpart, the majority of colonies included Colony Forming Unit-Granulocyte-Macrophage (CFU-GM) but also blast cell colonies and large numbers of colonies of Burst Forming Unit-Erythroids (BFU-Es). The number of colonies of PB34-ENU was also superior to the control (PB33), but no significant differences were observed with its unmutagenized counterparts (PB34) (Figure 4B). Interestingly, no BFU-Es were observed in the PB34, nor in the PB34-ENU condition; colonies were mainly of blast type.

Cells collected on day +14 of hematopoietic differentiation were then assayed in long-term culture-initiating cell (LTC-IC) experiments to determine their self-renewal potential. As can be seen in Figure 4, only PB32-ENU-derived hematopoietic cells generated a long-term culture potential with essentially blast cell colonies, whereas no LTC-IC potential could be obtained in their non-mutagenized counterparts (PB32), and in control PB33-derived cells. (Figure 4C). All colonies generated from PB32-ENU were CFU-GMSs and blast colonies. PB34 and PB34-ENU also showed colonies with a blastic and CFU-GM morphology, and the number of colonies from PB34-ENU showed no significant difference compared to PB34 (Figure 4D).

In addition to *BCR::ABL1*, PB34 also carried the mutation *JAK2 V617F* in the same clone as previously reported [28]. Progenitors derived from PB34 and its mutated counterparts were therefore tested for their sensitivity to JAK inhibitor Ruxolitinib, alone or in combination with Imatinib. For both cell lines, a decreased number of colonies was observed while treated with both Imatinib and Ruxolitinib, meaning that the ENU treatment did not modify the activity of Ruxolitinib (Supplementary Figure S5).

### 3.5. Transcriptomic Analysis of Hematopoietic Cells Derived from Mutagenized and Non-Mutagenized CML-iPSCs

**Figure 5:**
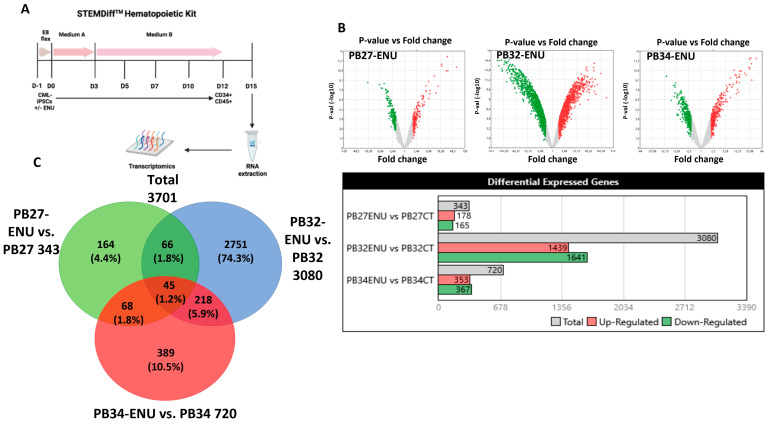
Transcriptomic profile comparison between three mutated CML-iPSCs and their normal counterparts after hematopoietic differentiation. (**A**) Hematopoietic differentiation protocol. Transcriptomic analysis was performed at D15 of differentiation. (**B**) Differential expression analysis (DEG) between ENU and control condition of each CML-iPSCs. (**C**) Venn diagram comparing the 3 DEG.

**Figure 6:**
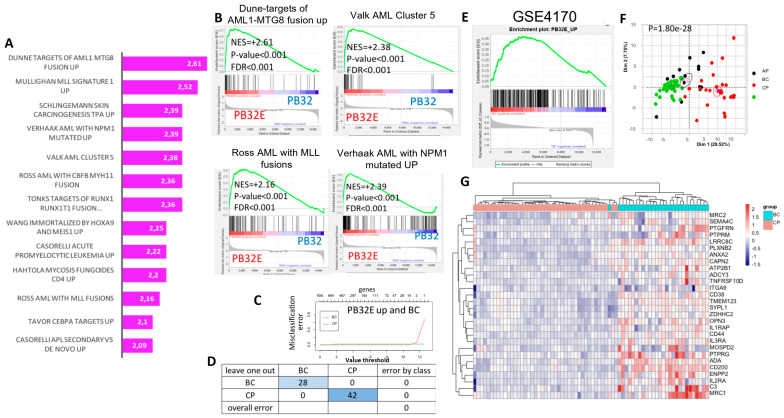
Blast-enriched signatures by ENU on PB32 CML-iPSCs differentiated into hematopoiesis (**A**) Bar plot of GSE analysis between ENU and control condition for PB32 performed on C MSigDb database. (**B**) Representation of AML GSE plot enriched signature on PB32 and PB32-ENU (PB32E). (**C**) Predictive signature for stratification of hematopoietic progenitors from CML patients in blast crisis as compared with ones from CML patients in the chronic phase. (**D**) Misclassification error. **(E)** Integration of PB32-ENU signature on GSE4170. (**F**) Unsupervised principal component analysis was performed with PB32-ENU-upregulated genes on HP of the distinct CML disease phase. (**G**) Upregulated receptors highlighted by integrative analysis during blast crisis.

Bulk transcriptomics analysis was performed from hematopoietic cells obtained at day +15 of the three distinct mutagenized or unmutagenized CML-derived iPSCs (Figure 5A). A differential gene expression was found between ENU and control condition for the individual three iPSCs allowing us to observe distinct expression profiles. Volcano plots (Figure 5B) after limma analysis highlighted a more important gene signature differentially expressed for the PB32 as compared to PB27 and PB34. These results are shown in Barplot and Venn diagram analyses (Figure 5B,C). Interestingly, we observed an enrichment of PB32-ENU signature with AML profiles, especially with AML5; this enrichment was observed only for PB32-ENU cells and not for their unmutated counterpart (Figure 6A,B). Genes upregulated of PB32 transcriptomics were used as a predictive signature for stratification of hematopoietic progenitors from BC-CML patients as compared with CML patients in CP. This learning machine process allowed us to clearly discriminate patient cells from the two distinct disease phases (Figure 6C) with null misclassification error (Figure 6D). These results suggest that ENU treatment on PB32 cells upregulated genes that reflect blast transformation of the advanced disease stage. GSEA analysis confirmed a significant enrichment of genes upregulated in PB32-ENU cells as compared to their non-mutagenized counterparts (Figure 6E). Unsupervised principal component analysis performed with PB32-ENU-upregulated genes on HP of the distinct CML phases, CP: chronic phase, AP: accelerated phase, and BC: blast crisis, showed a significant progressive separation of disease phases on the first principal axis (*p*-value = 1.8 × 10^−28^, Figure 6F). 

Functional enrichment performed with PB32-ENU-upregulated genes on Gene Ontology cellular component analysis was then allowed to identify some membrane markers present in this signature (Figure 6G), confirming the upregulation of these membrane markers in blast crisis as compared with chronic phase by unsupervised clustering (Figure 6G). Among these markers upregulated in BC-CML, *CD25* (alias *IL2RA*) was found to be of interest.

### 3.6. Single-Cell Transcriptomics during Hematopoietic Differentiation of ENU-Mutagenized iPSCs as Compared to Their Non-Mutagenized Counterparts

**Figure 7:**
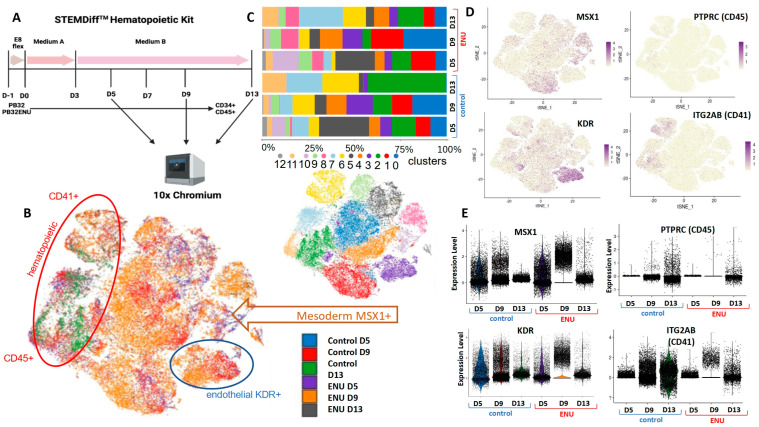
RNA Single-cell transcriptomics analysis of PB32 and PB32-ENU at D5, D9, and D13 of hematopoietic differentiation. (**A**) STEMDiff Hematopoietic differentiation protocol. Cells were analyzed at D5, D9, and D13. (**B**) tSNE analysis stratified on experimental conditions and cell population. (**C**) Bar plot of the percentage of cell population in each experimental condition. (**D**) tSNE analysis of relevant markers of mesoderm (MSX1), endothelium (KDR), and hematopoietic (CD45 and CD41). (**E**) Violin plots show gene expression of MSX1, PTPRC, CD45, and CD41.

To unravel the potential molecular events during the hematopoietic differentiation of mutagenized CML-iPSCs, we have chosen the PB32-ENU cell line to perform single-cell transcriptomics. Single-cell transcriptomics was processed by 10x genomics 3′ RNA technology on the PB32 cell line treated or not treated with ENU at day +5, day +9, and day +13 of hematopoietic differentiation (Figure 7A). The six individual experiments were integrated into a single-cell object composed of 63 432 cells after preprocessing. After tSNE dimension reduction, it could be observed that distinct cell repartition between cells of the distinct experimental conditions corresponds to 13 cell communities (Figure 7B). Evaluation of cell community proportions through the distinct experiments highlighted a reduction in the proportion of cell community number 2 (in green) in condition ENU D13 as compared with control day +13 cells (Figure 7C) corresponding to cells expressing *PTPRC* (alias *CD45*, adult hematopoietic marker) and *ITGA2B* (alias *CD41*, adult megakaryocyte marker) (Figure 7D) suggesting a lower proportion of differentiated hematopoietic cells in conditions treated by ENU as compared to controls. Indeed, *KDR* endothelial marker and *MSX1* mesodermal marker were found to be highly upregulated in the ENU day +9 condition as compared with the control condition (Figure 7E). *KDR* was found to be particularly expressed by cell community number 3 (Figure 7B,D), which is more represented by the ENU day +9 condition than by the control day +9 condition (Figure 7E). *MSX1* mesodermal marker is particularly expressed by clusters 1 and 8 (Figure 7B), and cluster 8 is mainly present in ENU conditions at distinct times (pink cluster, Figure 7D). Cluster 1 (red cluster), also expressing *MSX1*, is still present at D13 in the ENU condition but absent in D13 of the control condition (Figure 7E). Altogether, these results suggested that ENU treatment delayed hematopoietic differentiation of CML-derived iPSCs with a still accumulation of mesodermal precursor at day +13 of differentiation.

### 3.7. CD25 Is Overexpressed in BC-CML as Compared to CP-CML 

**Figure 8:**
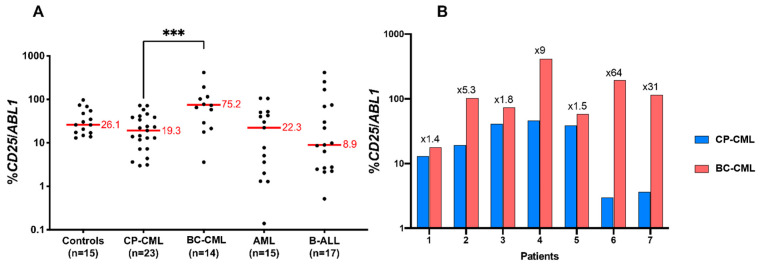
*CD25* expression in CP-CML, BC-CML, AML, and ALL patients. (**A**) *CD25* is overexpressed in BC-CML patients. The scatter dot plot shows the *CD25* mRNA expression in CML patients in the chronic phase at diagnosis and patients in the acute phase compared with healthy donors and patients with acute leukemias. For each group, the median is shown in red. *p*-value was calculated by the two-sided Mann–Whitney test. The three (***) asterisks indicate a *p* value < 0.001. (**B**) *CD25* mRNA levels are increased in all CML patients who progressed to an acute phase. The interleaved bar diagram shows the evolution of the *CD25* mRNA expression in 7 patients who were analyzed at the chronic and blast phases. *CD25* expression is shown in a logarithmic (Log10) scale, and the multiplication coefficient between the two phases is shown. Two data points are outside the axis limits (>1000).

Based on its important clinical relevance, we wished to evaluate the level of *CD25* expression in patients at different phases of CML. A cohort of 22 patients at CP-CML and 14 patients at BC-CML was analyzed for *CD25* expression using qRT-PCR as compared to *ABL* control (Table 3). For the experiments, we have also used blood RNA from normal controls (*n* = 15), CP-CML patients (*n* = 22), BC-CML (*n* = 14), AML (*n* = 15), and acute lymphoblastic leukemia (ALL) (*n* = 17). As can be seen in Figure 8A, as compared to the expression found in normal blood, CP-CML, and ALL, the expression of *CD25* was found to be increased in BC-CML (percentage of *CD25/ABL1* = 19.3 in CP-CML and 75 in BC-CML).

*CD25* mRNA expression was also evaluated comparatively in seven CML patients both in CP and after their evolution to BC-CML. *CD25* upregulation was clearly observed in all patients during BC-CML, with a 5- to 10-fold increase in four out of seven patients. (Figure 8B).

## 4. Discussion

BC-CML remains a life-threatening complication of CML and still occurs in the TKI era, especially in patients with TKI resistance. Modeling BC-CML is, therefore, an unmet need for developing new biomarkers in order to predict BC progression and discover new therapies. The goal of this work was to take advantage of the theoretically unlimited proliferation potential of iPSCs, to create a progression model of CML in vitro, using patient-derived iPSCs. We have previously shown the feasibility of generating CML-patient-derived iPSC using leukemic cells [27,28]. To model the blast crisis, which is due to the accumulation of genetic events in the context of *BCR::ABL1* expression, we chose the use of the alkylating agent ENU. We have previously shown that this procedure gives rise to one of the PB32 cell lines used here, a global genomic instability [26]. In this work, we further analyzed this event in three CML-iPSCs by generating two additional CML-iPSCs mutagenized by ENU treatment. CML-iPSCs treated with ENU exhibited increased levels of γ-H2AX phosphorylation, suggesting a DNA damage response (Supplementary Figure S1). To further evaluate the ENU-induced mutagenesis, we performed exome sequencing to uncover the mutations and their stability at the genetic level. Mutations identified in the ENU-treated condition confirm the mutagenic effect of ENU, and some of the affected genes are shown to be involved in the genomic instability in cancer (Figure 1). We then performed hematopoietic differentiation assays from ENU-mutagenized iPSCs as compared to their unmutagenized counterparts. In these experiments, the PB32-ENU cell line showed major and reproducible morphological changes, with in vitro generation of large numbers of blast cells indistinguishable from AML of monoblastic morphology (AML-5). These experiments were highly reproducible using this cell line (n = 10 experiments). These cells proliferated also in the MS-5 cell line but did not generate growth-independent cell lines, did not give rise to leukemia growth in NSG mice, and did not generate evidence of grafting (data not shown). Between the other two iPSCs lines, the “blast” type of transformation was not observed, but in the PB34-ENU cell line (expressing also *JAK2 V617F* mutation), we observed cytological changes with increased numbers of undifferentiated cells at day +12 and +14 of differentiation. In the PB27 cell line, although there was a clear transcriptomic difference between the ENU-mutagenized iPSC and its unmutagenized counterpart, we did not observe a cytological difference at day +12 of the cultures. It is most likely that these differences highlight not only patient-related genomic sensitivity to mutagenesis but also disease-related differences.

In the PB32-ENU-derived hematopoietic cells, we observed phenotypically exhibited myeloid cell surface markers such as MPO, CD34, CD45, CD33, and CD13. (Figure 3). A difference in the level of expression is an interesting indicator of the type of AML generated; for instance, low levels of CD34 for PB32 correspond to AML [44], which has also been found in the transcriptomic results (Figure 6). On the other hand, PB34-ENU exhibits high levels of CD34+ related to another type of AML (M0-M1) [45]. Meanwhile, both cell lines PB32-ENU and PB34-ENU gave rise to CD3-, CD79-, and CD19- cells, showing definitely their myeloid characteristics. In clonogenic assays, there was an increase in the number of colonies for the PB32-ENU compared to unmutagenized PB32 (more than ten-fold) and PB33 (Figure 4A). This increased number of colonies could reflect the undifferentiated status of mutated cells observed morphologically and phenotypically. The majority of colonies exhibited blast morphology, reinforcing the hypothesis that the increased number of colonies is directly related to the arrest of differentiation. PB34 and its mutated counterpart also showed an increased number of colonies compared to the control, but no significant difference was observed between the ENU-treated and untreated PB34. This might be related to the clinical background of the patient UPN-PB34, who was already in AP-CML and later developed a BC-CML (Table 1). Interestingly, no BFU-E were observed among the PB34, nor in PB34-ENU, there were all heterogenous with a CFU-GM and blast cell colony morphology. Another interesting characteristic was the important increase in the hematopoietic potential in PB32-ENU as assessed by LTC-IC (Figure 4C) assays. LTC-IC assays of PB34 harboring JAK2V617F mutation in addition to BCR::ABL [28] showed evidence of increased hematopoietic potential but no significant differences before and after mutagenesis (Figure 4D). The effect of Ruxolitinib alone or in combination with Imatinib on PB34 and PB34-ENU cells shows that ENU mutation does not affect the effectiveness of these drugs on the *JAK2 V617F* mutation (Supplementary Figure S5).

The transcriptional analysis confirmed that PB32-ENU exhibited the highest number of mutated genes followed by PB34-ENU and PB27-ENU (Figure 5B), and this was consistent with the potent BC-CML phenotype showed by PB32-ENU when compared to BC datasets (Figure 5B). PB32-ENU enrichment signatures showed an AML profile, whereas this is not the case for PB32. This shows the effect of the ENU on the in vitro blastic transformation of the disease. Comparing GSEA4170, which contains expression profiles of CML patients in all three different stages of CML, and the transcriptome of PB32-ENU shows the relevance of our model through its high correlation with transcription profiles of patients in BC-CML (Figure 6E). This correlation with BC-CML patients allowed us also to discover the expression in PB32-ENU of a group of genes also expressed in patients in BC-CML (Figure 6G) and among which some are potential targets such as *IL2RA (CD25).* CD25 has already been described in AML blasts, BC-CML, and LSCs [46,47] as an aberrant marker and related to bad prognosis; it is also a potential target for new therapies in BC-CML and AML. 

To uncover the mechanisms of the generation of mutagenized hematopoietic cells from iPSCs, we have performed single-cell transcriptomics in the most ‘performant’ CML-iPSC line, PB32, during hematopoietic differentiation. For this experiment, we analyzed single cells at three different time points in order to better understand the effect of ENU during differentiation and the cell populations affected by the mutagenesis. Single-cell data reinforced the results we had obtained previously by confirming the hypothesis for a delay of differentiation due to ENU treatment. We could observe a change in different communities of cells between mutated and unmutated cohorts, as well as different genes. Among these genes, the most striking was the reduction in *CD45* (a marker of differentiated hematopoietic cells) and *CD41* (a marker of megakaryocytes) in the ENU-treated cells compared to untreated ones, suggesting strongly the arrest of hematopoietic differentiation observed morphologically and phenotypically. Moreover, the increase in *MSX1* (a marker of mesoderm) and *KDR* (a marker of hemangioblast) in ENU-treated cells but not in untreated cells reveals the accumulation of mesodermal and primitive markers and, consequently, the arrest of differentiation. 

After the identification of *CD25* expression in PB32-ENU and the correlation with the cohort of CML patients, we wished to evaluate the real amount of its expression during the progression of CML toward an AP/BC phase. Using a large number of CP-CML (*n* = 22) progressing toward BC-CML (*n* = 14) and AML cases, we have shown that the expression of *CD25* is increased during the progression of CML, and that this could be a potential therapeutic target (Figure 8A,B). Other targets that we have identified are currently under study. 

Overall, these experiments validate the relevance of our model for analysis of CML progression using iPSCs due to their major proliferation potential and their potential of use as “off-the-shelf” tools for individual CML patients but also in other progressive hematological malignancies such as myelodysplastic syndromes (MDS) and AML. 

These studies also present some limitations. The clinical background of the patients and their heterogeneity are certainly a limitation, and these experiments need to be extended to other CML-iPSCs, which might validate this interesting tool to predict the probability of CML progression in patients. In the current study, the validation of discovered markers in patients is proof of the representativeness of our model, and it can be used for drug screening or novel drug discovery.

## 5. Conclusions

CML-iPSCs represent a novel and disruptive technology for modeling the pathophysiology of CML and for discovering novel targets. ENU-induced in vitro mutagenesis allows the generation of a dynamic, in vitro model of CML progression, allowing the discovery of relevant biomarkers and novel targets. Several novel targets identified are now under study.

## Figures and Tables

**Table 1:** Clinical characteristics of the patients.

Patient #	Age/Sex	Status at Diagnosis	Reprogrammed Cells/Origin
UPN27 (PB27)	56/M	CP-CML	CD34+ / Blood
UPN32 (PB32)	17/M	CP-CML	CD34+ / Blood
UPN34 (PB34)	70/M	CP-CML	CD34+ AP-CML/Blood

(CP-CML: Chronic Phase CML; PB: Peripheral Blood).

**Table 2:** Antibody fluorophores and references.

Antibody	Fluorophore	Reference
CD45	PC7	IM3548 Beckman Coulter
CD13	PE	A07762 Beckman Coulter
CD33	APC	IM2471 Beckman Coulter
CD117	PC5.5	B96754 Beckman Coulter
MPO	FITC	IM1874 Beckman Coulter
CD19	ECD	6604551 Beckman Coulter
CD43	PE	A32560 Beckman Coulter
CD3	APC-A750	B10823 Beckman Coulter
CD22	PC5.5	A80712 Beckman Coulter
CD79a	APC	B36287 Beckman Coulter
HLA-DR	ECD	B92438 Beckman Coulter
CD34	APC-A70	B92417 Beckman Coulter

**Table 3:** **Characteristics of the different cohorts tested for CD25 mRNA expression.** CP-CML, chronic phase CML; BC-CML, Blast crisis CML; AML, acute myeloid leukemia; B-ALL, B-cell lymphoblastic leukemia; N/A, anonymized healthy donors.

	*Controls*	*CP-CML*	*BC-CML*	*AML*	*B-ALL*
n	15	23	14	15	17
Gender					
Male	*N/A*	13	11	9	5
Female	*N/A*	10	3	6	12
Age, range (years)	*N/A*	58.1 [22.7–81.1]	47.4 [22.9–75.2]	67.6 [26.0–83.6]	37.8 [4.6–82.5]
**Molecular Rearrangements**					
*BCR::ABL1* *M-BCR (e13a2 or e14a2)*		23	14		
*BCR::ABL1* *m-BCR (e1a2)*					5

## Data Availability

Not applicable.

## Supplementary Materials

The following supporting information can be downloaded at: https://www.mdpi.com/article/10.3390/cells12040598/s1. Supplementary Figure S1: Generation of ENU mutagenized CML-iPSC. Supplementary Figure S2: Pluripotency confirmation of mutagenized and unmutagenized CML-iPSC with ENU. Supplementary Figure S3: Morphological changes observed during hematopoietic differentiation of ENU mutagenized and unmutagenized CML iPSC and MGG staining at D12 of differentiation. Supplementary Figure S4: Imatinib and Ruxolitinib sensitivity assay. Supplementary Figure S5: Imatinib and Ruxolitinib sensitivity assays in PB34 CML iPSC expressing both BCR:ABL and JAK2V617F mutation.
