# Supplementary material for: Modeling Blast Crisis Using Mutagenized Chronic Myeloid Leukemia-Derived Induced Pluripotent Stem Cells (iPSCs)
Source: Cells. 2023 Feb 12;12(4):598. doi: 10.3390/cells12040598 (PMC9953961; doi:10.3390/cells12040598)
Supplement: Supplementary file 1 [file cells-12-00598-s001.zip › cells-2161111-supplementary.pdf]

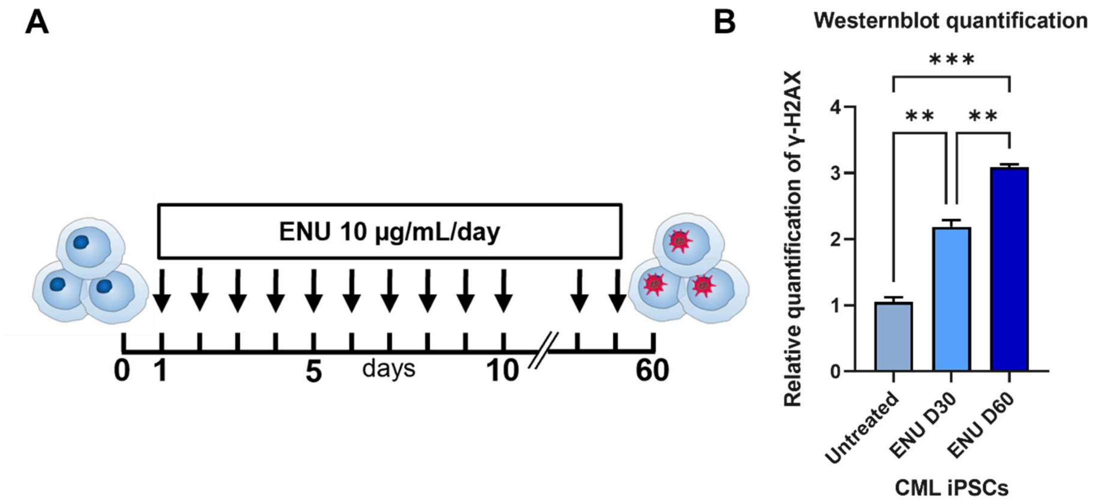

Supplementary Figure S1: Generation of ENU mutagenized CML-iPSCs.

(A) Schematic representation of CML-iPSC mutagenesis with ENU. Arrows show the daily treatment with ENU for 60 days. **(B)** Relative quantification of γ-H2AX in ENU-treated CML-iPSC at day 30 and day 60 using western blot analysis. Phospho γ-H2AX was normalized to b-actin and control. Mean of 3 CML iPSCs treated with ENU were compared and P-values were calculated using two-tailed Student's t-test. **, $P < 0.01$; ***, $P < 0.001$.

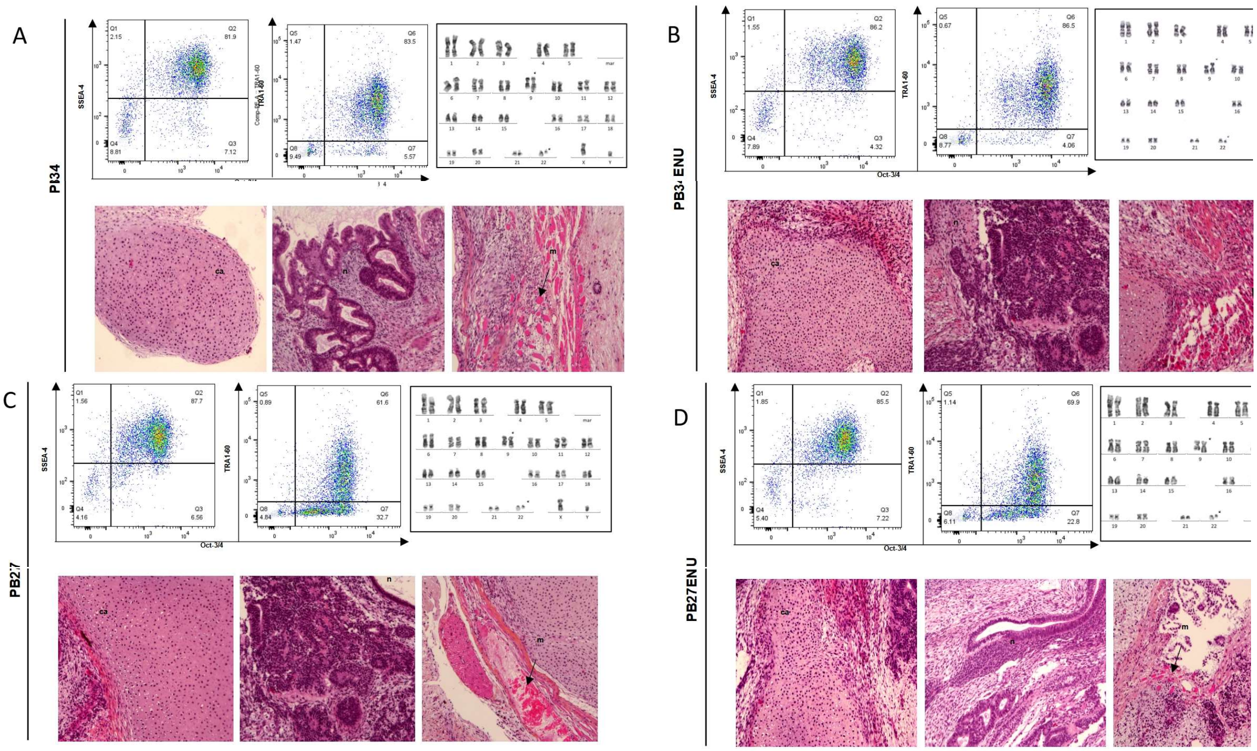

Supplementary Figure S2: Pluripotency confirmation of mutagenized and unmutagenized CML-iPSCs with ENU.

Pluripotency was tested by flow cytometry, teratoma induction, karyotype checking for absence of chromosomal aberrations and presence of Ph+ chromosome for PB34 **(A)**, PB34-ENU **(B)**, PB27 **(C)**, PB27-ENU **(D)**. ca: Cartilage; M: muscle; n: Neural crest.

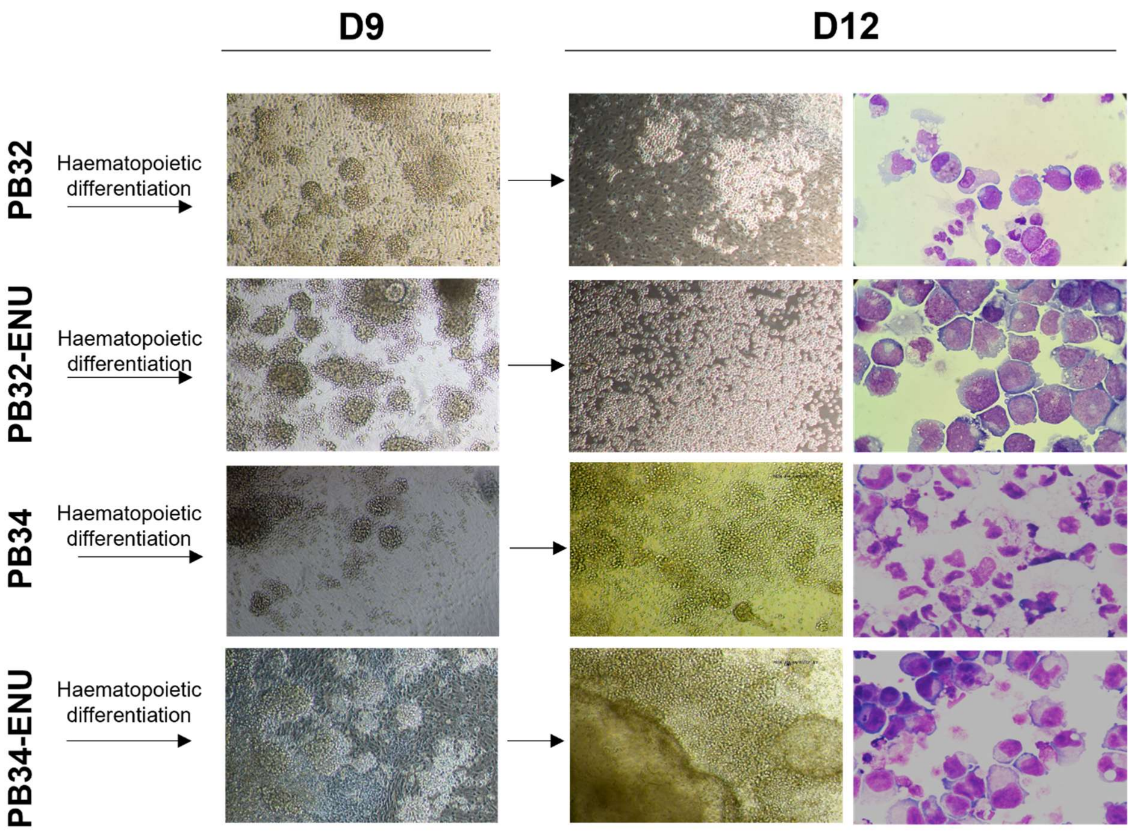

Supplementary Figure S3: Morphological changes observed during hematopoietic differentiation of ENU mutagenized and unmutagenized CML-iPSCs and MGG staining at D12 of hematopoietic differentiation.

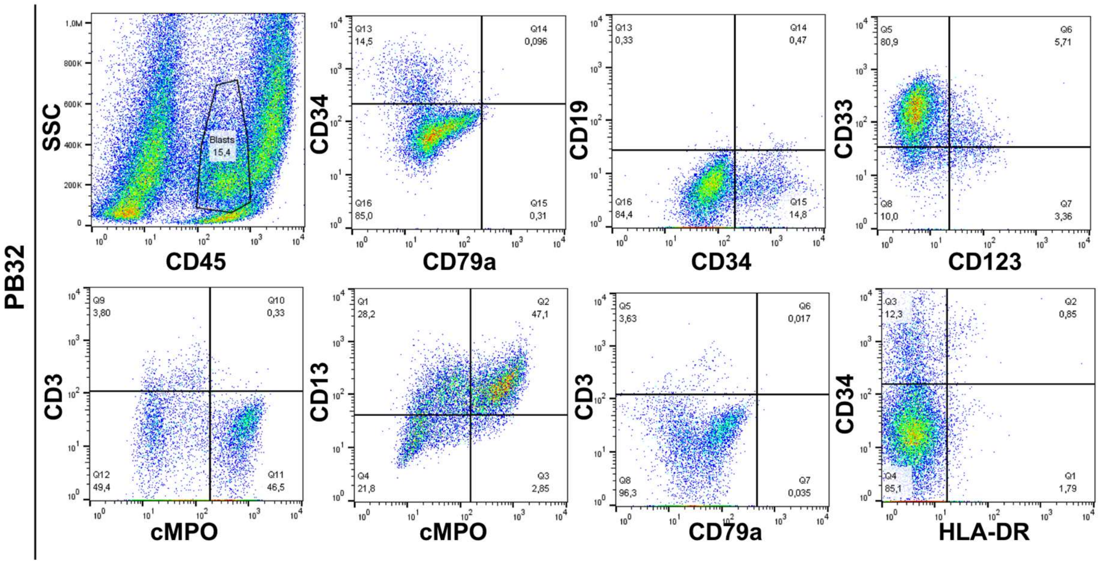

Supplementary Figure S4: Phenotypic characterization of non-mutagenized BCR-ABL-expressing PB32 cell line.

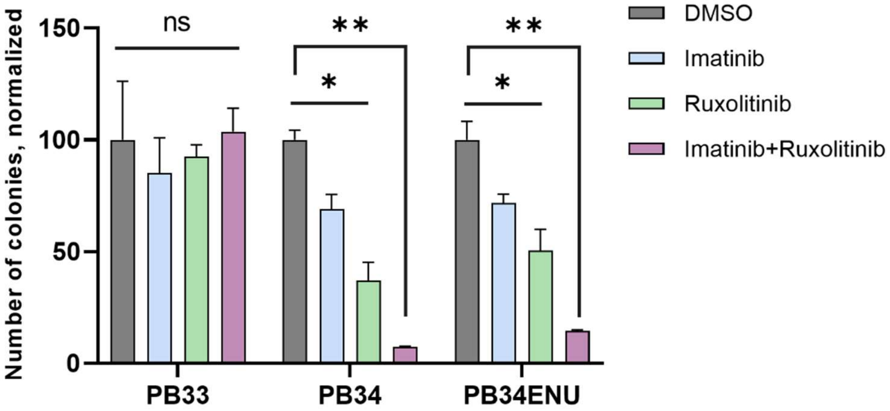

Supplementary Figure S5: Imatinib and Ruxolitinib sensitivity assays in PB34 CML iPSC expressing both BCR::ABL and JAK2V617F mutation.

Number of colonies normalized to control (PB33) after incubation either with Imatinib, Ruxolitinib or combined for PB34 and PB34-ENU hematopoietic derived cells. Mean number of colonies treated with TKIs were compared to control DMSO for each CML-iPSC and P-values were calculated using two-tailed Student's t-test. ns, not significant; *, $P < 0.05$.**, $P < 0.01$.
